# Investigating the susceptibility of treatment-resistant oesophageal tumours to natural killer cell-mediated responses

**DOI:** 10.1007/s10238-022-00811-6

**Published:** 2022-04-01

**Authors:** Eimear Mylod, Ellen McKenna, Maria Davern, Martin P. Barr, Noel E. Donlon, Becky A. S. Bibby, Anshul Bhardwaj, John V. Reynolds, Joanne Lysaght, Stephen G. Maher, Melissa J. Conroy

**Affiliations:** 1 Cancer Immunology and Immunotherapy Group, Department of Surgery, Trinity Translational Medicine Institute and Trinity St. James’s Cancer Institute, St. James’s Hospital, Trinity College Dublin, Dublin 8, Ireland; 2 Thoracic Oncology Research Group, Trinity Translational Medicine Institute and Trinity St. James’s Cancer Institute, St. James’s Hospital, Trinity College Dublin, Dublin 8, Ireland; 3 Translational Radiobiology Group, Division of Cancer Sciences, University of Manchester, Manchester Academic Health Science Centre, Christie Hospital, Manchester, M20 4BX UK; 4 Department of Surgery, Trinity Translational Medicine Institute and Trinity St. James’s Cancer Institute, St. James’s Hospital, Trinity College Dublin, Dublin 8, Ireland; 5 National Oesophageal and Gastric Centre, St. James’s Hospital, Dublin, Ireland

**Keywords:** NK cells, Oesophageal adenocarcinoma, NK cell receptors, Treatment-resistance, Immunotherapy

## Abstract

The majority of oesophageal adenocarcinoma (OAC) patients do not respond to multimodal treatment regimens and face dismal survival rates. Natural killer (NK) cells are crucial anti-tumour immune cells, and this study investigated the susceptibility of treatment-resistant OAC cells to these potent tumour killers. Natural killer receptor (NKR) ligand expression by OE33CisP (cisplatin-sensitive) and OE33CisR (cisplatin-resistant) cells was investigated. The immunomodulatory effects of OE33CisP and OE33CisR cells on NK cell phenotype and function were assessed. Finally, the impact of chemotherapy regimens on NKR ligand shedding was examined. Our data revealed significantly less surface expression of activating ligands B7-H6, MICA/B, ULBP-3 and activating/inhibitory ligands PVRL-1 and PVRL-4 by OE33CisR cells, compared to OE33CisP cells. Co-culture with OE33CisR cells reduced the frequencies of NKp30^+^ and NKp46^+^ NK cells and increased frequencies of TIGIT^+^, FasL^+^ and TRAIL^+^ NK cells. Frequencies of IFN-*γ*-producing NK cells increased while frequencies of TIM-3^+^ NK cells decreased after culture with OE33CisP and OE33CisR cells. Frequencies of circulating NKp30^+^ NK cells were significantly lower in OAC patients with the poorest treatment response and in patients who received FLOT chemotherapy, while B7-H6 shedding by OAC tumour cells was induced by FLOT. Overall, OE33CisR cells express less activating NKR ligands than OE33CisP cells and have differential effects on NKR expression by NK cells. However, neither cell line significantly dampened NK cell cytokine production, death receptor expression or degranulation. In addition, our data indicate that FLOT chemotherapy may promote B7-H6 shedding and immune evasion with detrimental consequences in OAC patients.

## Introduction

Oesophageal adenocarcinoma (OAC) is an aggressive malignancy with a dismal 5-year survival rate of ~ 19%, placing it as the sixth highest cause of cancer-related death globally [1]. Late presentation of disease and low treatment response rates in OAC greatly contribute to its poor prognosis. For locally advanced OAC, the current standard-of-care is chemo-radiotherapy (CRT) and surgical resection, however, only ~ 30% of these patients have a complete response, meaning that ~ 70% are resistant to standard-of-care therapies.

As immunotherapies begin to change the landscape of OAC, there is space for cellular based immunotherapies to significantly contribute to this field. With the recent approval of immune checkpoint inhibitor nivolumab plus chemotherapy as a first line treatment for advanced gastric cancer, it is evident that immunotherapies are beginning to make an impact in this space [2]. However, immune checkpoint inhibitor efficacy in gastric cancer stands at 22%, meaning there is a large population of patients who do not gain clinical benefit from currently available immunotherapies [3]. As such, a sizeable population of patients may still be exposed to standard-of-care chemotherapies, such as cisplatin, as a first-line treatment.

Natural Killer (NK) cells are innate lymphocytes that play a critical role in tumour eradication and it is well-established that their dysfunction in cancer is associated with poorer patient outcomes [4]. Activated NK cells are potent cytokine producers and elicit powerful cytotoxic activities to induce tumour cell death via perforin/granzyme-mediated-mechanisms, Fas ligand (FasL) and TNF-related apoptosis-inducing ligand (TRAIL) [5–7]. Unlike T cells, NK cells do not require specific antigen recognition via the major histocompatibility complex (MHC) and instead become activated through a fine balance of ligation with their inhibitory and activating receptors [8]. NK cell receptors (NKRs) include the NK group 2 (NKG2) family of receptors, such as the NKG2A inhibitory and NKG2D activating receptors and the activating natural cytotoxicity receptors (NCR), natural killer cell p30-related protein (NKp30) and NKp46 [8].

Adoptive T cell-based therapies have been at the fore of cellular immunotherapies for cancer and have been FDA-approved for multiple blood malignancies [9]. However, T cells have a number of perceived disadvantages compared to their innate lymphocyte counterparts in NK cells, including the requirement for antigen specificity and the requirement to be sourced autologously [10]. Indeed, this limits their applicability and scalability, precluding them from becoming a truly off-the-shelf immunotherapeutic option [10]. As such, NK cells present themselves as an adept alternative to T cells as a mediator of cellular based immunotherapies and have been explored extensively [11].

To our knowledge, we have characterised for the first time, the NKR ligand profiles and immunomodulatory properties of chemotherapy-resistant and chemotherapy-sensitive OAC tumour cells. Importantly, we have examined the susceptibility of chemotherapy-resistant OAC tumours to NK cell-mediated responses and provide novel insights into the amenability of NK cell therapies for a patient cohort of whom 70% derive no clinical benefit from current treatment modalities.

## Materials and methods

### Cell culture

The OE33 oesophageal adenocarcinoma cell line was purchased from the European collection of cell cultures. Cisplatin-sensitive (OE33CisP) and cisplatin-resistant (OE33CisR) OE33 cells were derived from the original parent OE33 cell line as previously described [12, 13]. OE33, OE33CisP and OE33CisR cells were cultured in RPMI 1640 supplemented with 2 mM L-glutamine, 10% foetal bovine serum and 1% penicillin–streptomycin (Gibco) at 37 °C in a humidified atmosphere of 5% CO_2_. Cells were routinely mycoplasma tested. Isogenic OE33CisP and OE33CisR cell lines were age and passage matched.

### Surface and intracellular staining for flow cytometric analysis

OE33CisP and OE33CisR cells were stained for flow cytometry using PVRL-1-AlexaFlour488, PVRL-4-PE, ULBP-3-APC, B7-H6-PE (R&D, USA), MICA/B-PE-Vio770, HLA-E-FITC, 4-1BBL-PE-Vio770, PVR-APC (Miltenyi Biotec) and TRAIL-R2-PE (Biolegend).

For co-culture, OE33CisP and OE33CisR cells were trypsinised at 80% confluency and seeded at a density of 2 × 10^5^ cells/ml in RPMI supplemented with 10% FBS and 1% penicillin–streptomycin (Gibco). Peripheral blood mononuclear cells (PBMC) were isolated from the blood of healthy donors by density gradient centrifugation and resuspended in RPMI supplemented with 10% FBS and 1% penicillin–streptomycin (Gibco). PBMC were co-cultured at a 1:1 ratio with OE33CisP or OE33CisR cells for 24 h.

Following co-culture, PBMC were stained for flow cytometry using the following surface antibodies; CD56-FITC-Viobright, NKG2A-APC (Miltenyi-Biotec) CD3-APC-Cy7, NKG2D-PE-Cy5, NKP46-PE-Cy7, NKp30-BV421, CD69-BV510, TIGIT-PE-Cy5, PD-1-PE-Cy7, TRAIL-APC, FasL-BV421, LAG3-PE-Cy7, CTLA-4-PE-Cy5 (Biolegend), A2AR-PE (Bio-techne) and TIM-3-AF647 (BD Biosciences).

For intracellular staining, cells were stimulated with 10 ng/ml PMA and 1 µg/ml Ionomycin for a total of four hours. CD107a (Biolegend) was added at this time to detect degranulation. After one hour, 10 µg/ml of Brefeldin A (Sigma) was added for the remaining three hours. Cells were stained for surface markers with CD56-FITC-Viobright (Miltenyi-Biotec) and CD3-APC-Cy7 (Biolegend). Cells were stained for intracellular markers with TNF-*α*-APC, IL-10-BV421 and IFN-*γ*-BV510 (Biolegend) using the FIX&PERM Cell Fixation and Permeabilization Kit (Nordic MUBio).

For the quantification of NKP30^+^ NK cells in OAC patient blood, whole blood was collected at a time point of surgical resection and was stained with CD56-FITC-Viobright (Miltenyi Biotec), CD3-APC-Cy7 and NKp30-BV421 (Biolegend). Red blood cells were lysed using BD Lysing Solution (BD Biosciences) as per manufacturer’s instructions. NK cells were quantified as CD56^+^CD3^−^ cells within the lymphocyte gate.

All samples were acquired using the BD FACS CANTO II (BD Biosciences) flow cytometer and analysed using FlowJo v10 software (Tree Star). NK cells were defined as CD56^+^CD3^−^ in the lymphocyte gate.

### Treatment of OE33 cells with clinically relevant combinations of chemotherapy

OE33 cells were seeded at a density of 5,000 cells/200 µl media and treated for 48 h with the chemotherapeutics from the FLOT (0.8249 µM 5-fluorouracil, 2 µM oxaliplatin, 0.001 µM docetaxel), CROSS (0.001 µM paclitaxel, 1000 µM carboplatin, ± 2 Gy irradiation) or MAGIC (1.493 µM epirubicin, 1.5 µM cisplatin, 0.8249 µM 5-flurouracil) (Sigma) regimens to simulate the effects of the most commonly used chemotherapeutic regimens for OAC patients. An IC_25_ of each treatment was used which resulted in 50% of OE33 cell viability, as determined by a CCK8 assay. Following treatment, supernatants were collected.

### Quantification of soluble NKR Ligands in the OAC microenvironment and OAC patient serum

OE33CisP and OE33CisR cells were pelleted and supernatants were collected and cryopreserved at -80 °C. Supernatants from parent OE33 cells treated with clinically relevant regimens of chemoradiotherapy (CRT) as described above were collected and cryopreserved at -80 °C. Human B7-H6 duo-set ELISA, human ULBP-3 duo-set ELISA (R&D) and human MICA ELISA (ELISA Genie) were carried out as per manufacturer’s instructions. For normalisation, total protein was determined from all cell lines using the Pierce™ BCA Protein Assay Kit (ThermoFisher) as per manufacturer’s instructions. Absorbance values were measured at 595 nm using a VersaMax plate reader and SoftmaxPro 6.1 software from which a standard curve was generated to deduce total protein concentrations.

### Quantitative real-time PCR

Total RNA was isolated from cell lines using TRI Reagent (Molecular Research Center). RNA yield and purity were determined using a Nanodrop-1000 spectrophotometer. Gene expression analysis of the NK cell ligands PVRL-1, PVRL-4, HLA-E, B7-H6 and ULBP3, was carried out in duplicate using the Luna Universal One-Step RT-qPCR Kit (New England BioLabs Inc), in duplicate, on the ABI 7500 qPCR system. ß-actin was used to normalise for endogenous gene expression. Complementary DNA (cDNA) was generated by reverse transcription for 10 min at 55 °C. Real-time PCR was carried out by denaturation at 95 °C for 1 min followed by 45 cycles consisting of denaturation at 95 °C for 10 secs, primer extension at 60 °C for 60 secs and a final elongation step at 60 °C for 1 min. Mean CT values were determined and differences in gene expression levels were calculated as fold-changes relative to controls. Primers were designed using the University of California Santa Cruz (UCSC) Genome Browser and synthesised commercially (Sigma-Aldrich). Primers sequences used are as follows: PVRL-1 FWD 5’-TCTCGGCTTGACCGCATTCT-3’, REV 5’-TGGTGGACTTCTGCCATGTGAC-3’; PVRL-4 FWD 5’-GGAAGACTCTGGGAAGCAGG-3’, REV 5’-GATGGAGTTCTCCCTGGTCAG-3’; HLA-E FWD 5’-AAGACACATGCGTGGAGTGG-3’; REV 5’-TGTGATCTCCGCAGGGTAGA-3’; B7-H6 FWD 5’-TCCATTCATTGGTGGCCTAT-3’, REV 5’-TGCAAAAGAATATGAGGTGCTCT-3’; ULBP3 FWD 5’-GGCGGATGAAAGAGAAGTGG-3’, REV 5’-TTGGGTTGAGCTAAGCCTGG-3’, β-actin FWD 5’- TGTTTGAGACCTTCAACACCC-3’, REV 5’- AGCACTGTGTTGGCGTACAG-3’.

### Patient demographics

Blood sera and whole blood were collected from a total of 30 consenting OAC patients attending the National Oesophageal and Gastric Centre at St. James’s Hospital, Dublin (Table 1). The patient group included 22 males and 8 females, reflecting the predominance of OAC in males. The group ranged in age 48–83 years and had a mean age of 65.2 years. Within this cohort, 80% had received neoadjuvant CRT regimens. Tumour regression grade (TRG) was used to categorise patients as good responders (TRG 1–2) or poor responders (TRG 3–5) to chemo-radiotherapy [14]. The computed tomography defined visceral fat area (VFA) was calculated as described previously and categorised as obese when VFA was greater than 160cm^2^ for men and 80cm^2^ for women [15]. The mean VFA was 145.38cm^2^, with 55% of patients measuring as viscerally obese.

**Table 1:** Patient demographic table

Age (years) (range)	65.2 (48–83)
Sex ratio (M:F)	22:8
*Tumour stage*^1^	
T0	5
T1	5
T2	3
T3	12
T4	3
*Nodal status*^1^	
Positive	16
Negative	12
*Neo-adjuvant chemotherapy regimen*	
FLOT	15
CROSS	7
MAGIC	1
FOLFOX	1
*Tumour regression grade*^2^	
1–2	8
3–5	13
Mean VFA (cm^2^)^3^	145.38
Viscerally obese by VFA^3^	55%

^1^Tumour stage and Nodal status unknown for 2 patients

^2^Tumour regression grade unknown for 9 patients

^3^Obese VFA > 160 cm^2^ for men and > 80 cm^2^ for women (Doyle et al. 2013)

### Statistical analysis

Statistical analysis was performed using GraphPad Prism version 9 (GraphPad software). The statistical significance between groups was determined by a t test or one-way ANOVA with post-hoc Dunnett’s or Tukey’s test as appropriate. *P* < 0.05 was considered significant.

## Results

### NKR ligands are expressed at significantly lower levels by OE33CisR cells compared to OE33CisP cells

**Fig. 1:**
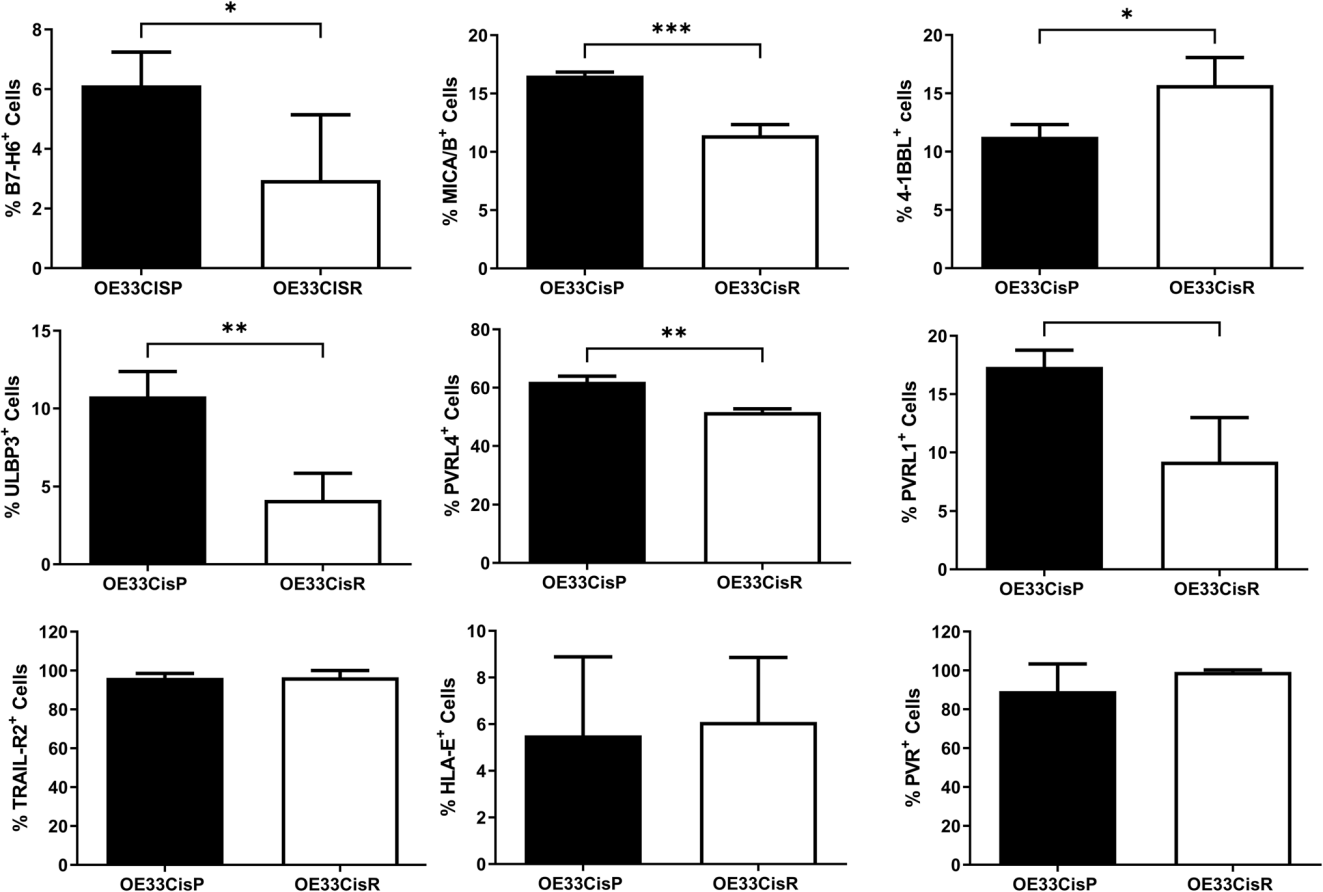
Significantly lower frequencies of OE33CisR cells expressing B7-H6, MICA/B, ULBP-3, PVRL-4 and PVRL-1 on their surface compared to OE33CisP cells. Bar chart showing the frequencies of OE33CisP and OE33CisR (*n* = 3–5) expressing NKR ligands B7-H6, MICA/B, 4-1BBL, ULBP-3, PVRL-4, PVRL-1, TRAIL-R2, HLA-E and PVR. *p < 0.05, ***p < 0.001 by unpaired t-test. [Mean + SEM]

The expression of NKR ligands on treatment-resistant OAC tumour cells was quantified to assess the susceptibility of treatment-resistant OAC tumour cells to NK cells. Here we report significantly lower frequencies of B7-H6^+^ (OE33CisP vs. OE33CisR; 7.544% vs. 2.955%; *p* = 0.049), MICA/B^+^ (OE33CisP vs. OE33CisR; 16.53% vs. 11.43%; *p* = 0.0008) and ULBP-3^+^ (OE33CisP vs. OE33CisR; 10.78% vs. 4.150%; *p* = 0.0080) cells within the OE33CisR population compared to the OE33CisP cell population (all *n* = 3) (Fig. 1). Additionally, significantly lower frequencies of cells expressing the activation/inhibitory ligands PVRL-4 (OE33CisP vs. OE33CisR; 62.03% vs. 51.60%; *p* = 0.0013) and PVRL-1 (OE33CisP vs. OE33CisR; 17.33% vs. 9.233%; *p* = 0.0252) (Fig. 1) were observed on the surface of OE33CisR cells, compared to OE33CisP cells.

In contrast, there were significantly lower proportions of OE33CisP cells expressing 4-1BBL (OE33CisP vs. OE33CisR; 11.28% vs. 15.07%; *p* = 0.0144) (Fig. 1) compared to OE33CisR cells. There were no significant differences in the frequencies of OE33CisP and OE33CisR cells expressing TRAIL-R2, HLA-E or PVR (Fig. 1).

### There are no significant differences in mRNA expression of NKR ligands between OE33CisR and OE33CisP cells

**Fig. 2:**
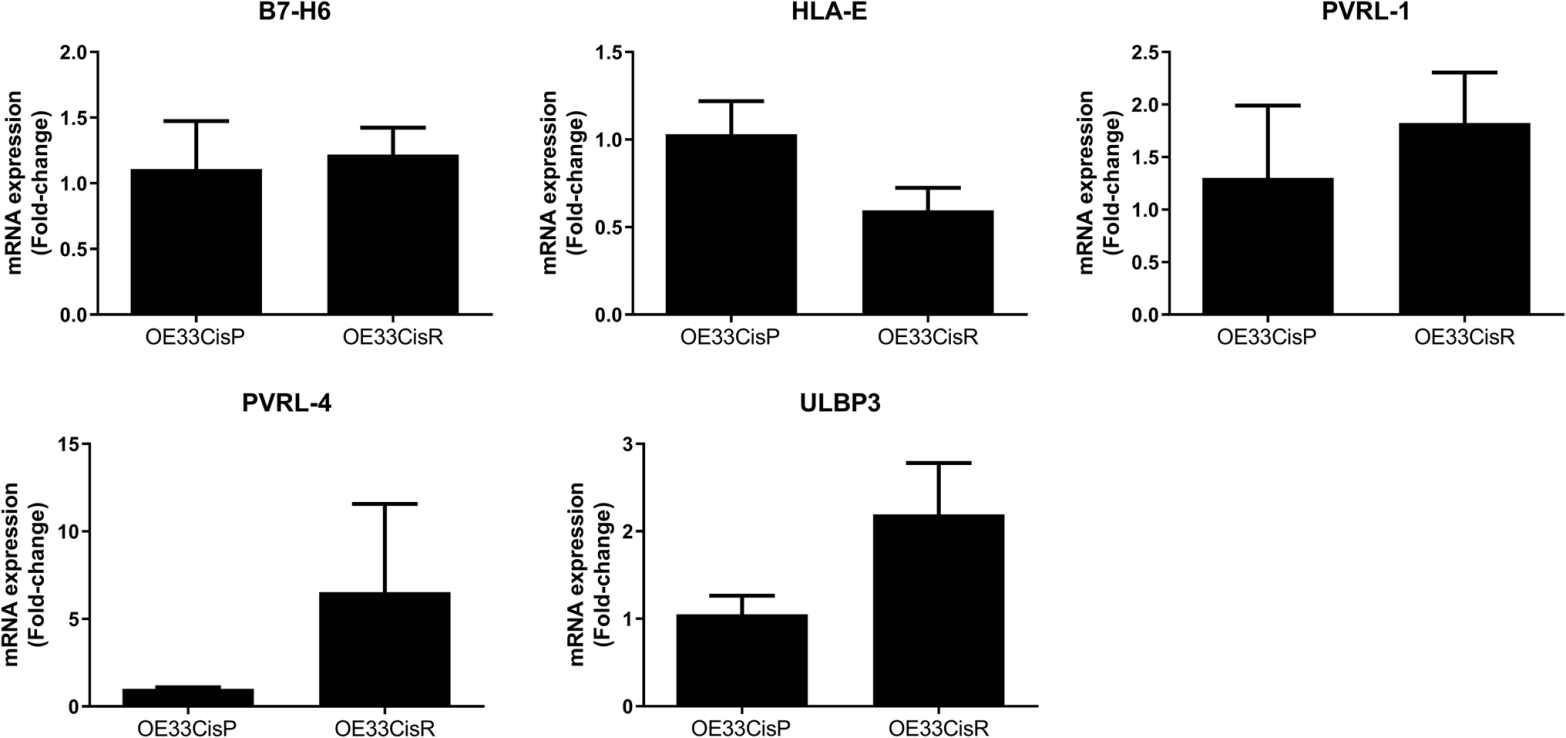
No significant differences in NK cell ligand mRNA expression between OE33CisP and OE33CisR cells. Bar chart showing the mRNA expression as fold change of the NK cell ligands (**A**) B7-H6, (**B**) HLA-E, (**C**) PVRL-1, (**D**) PVRL-4 and (**E**) ULBP3 in OE33CisP (left) and OE33CisR (right). Gene expression for each gene is normalised to the housekeeping gene, β-actin. Unpaired t-test [Mean + SEM]

To ascertain whether significantly lower surface expression of activating NKR ligands on OE33CisR cells was due, at least in part, to a downregulation of the genes encoding these ligands, mRNA levels were measured by RT-PCR. There were no significant differences in the expression levels of B7-H6, HLA-E, PVRL-1, PVRL-4 or ULBP-3 between OE33CisP and OE33CisR cells (Fig. 2). There was a trend however towards higher levels of PVRL-4 and ULBP-3 mRNA expression in OE33CisR cells relative to their parent counterparts.

### Standard-of-care chemotherapeutic regimens for OAC can modulate B7-H6 shedding by OE33 cells

**Fig. 3:**
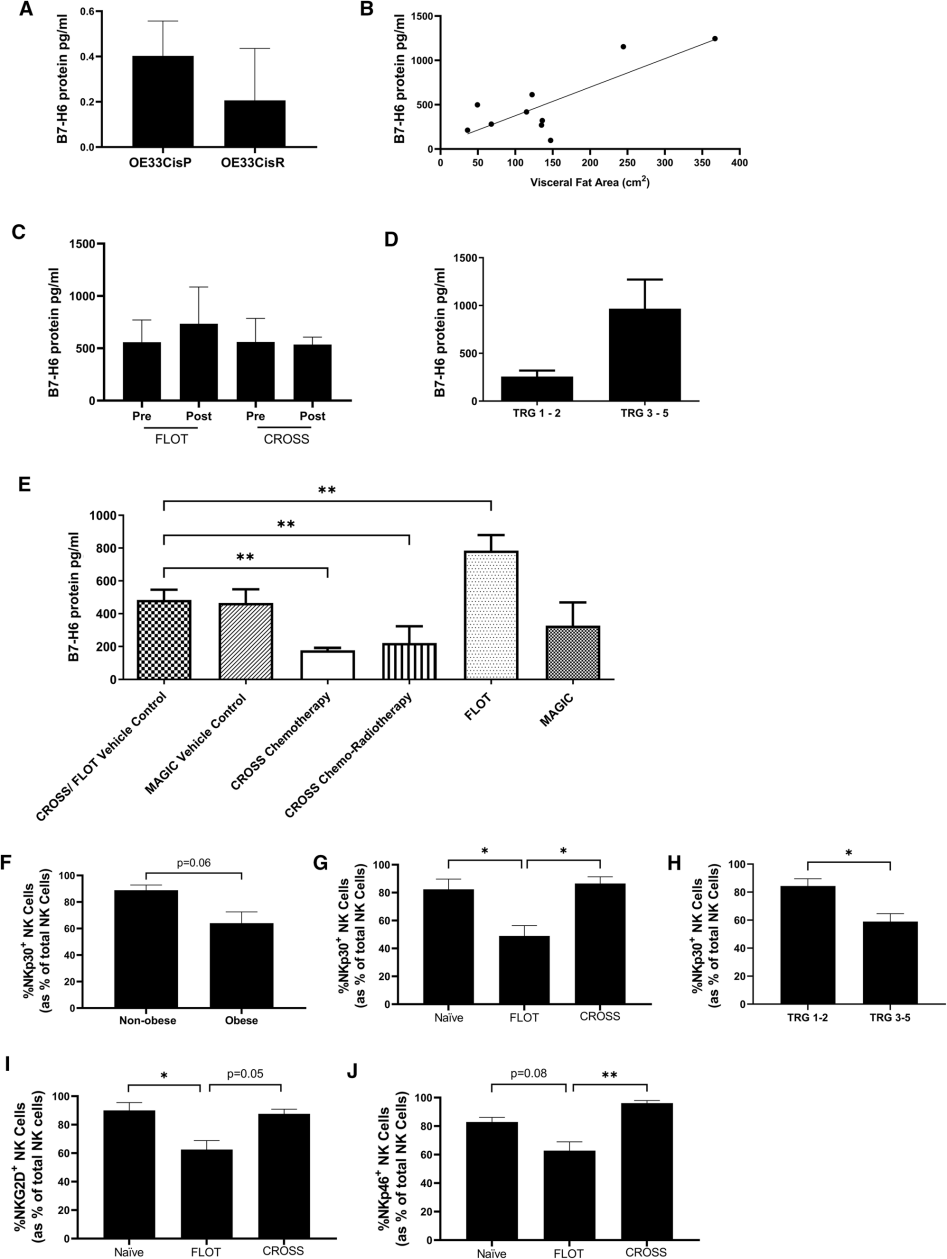
Differential levels of B7-H6 shedding and NKp30 surface expression in OAC patients following first-line treatments. (**A**) Bar chart showing the concentration of B7-H6 in OE33CisP and OE33CisR (*n* = 3) cell supernatant, normalised to protein content (µg/ml) (**B**) Line graph showing correlation between visceral fat area (VFA) and B7-H6 protein levels in serum of 10 OAC patients. (**C**) Bar chart showing B7-H6 concentration in the serum of OAC patients pre-FLOT (*n* = 10) (0.8249 M 5-fluorouracil, 2 µM oxaliplatin, 0.001 µM docetaxel) and post-FLOT (*n* = 5) (BLACK) and pre-CROSS (*n* = 4) (0.001 µM paclitaxel, 1000 µM carboplatin, 2 Gy irradiation) and post-CROSS (*n* = 2) (WHITE). (**D**) Bar chart showing B7-H6 concentration in the serum of OAC patients pre-treatment, stratified by tumour regression grade TRG 1–2 (*n* = 4, good responders) (BLACK) and TRG 3–5 (*n* = 5, poor responders) (WHITE) *n* = 5). (**E**) Bar chart showing the concentration of B7-H6 (pg/ml) in OE33 cell supernatant following treatment with vehicle control, CROSS chemotherapy only, CROSS chemoradiotherapy (CRT), FLOT chemotherapies, or MAGIC chemotherapy (all *n* = 3). FLOT (0.8249 M 5-fluorouracil, 2 µM oxaliplatin, 0.001 µM docetaxel), CROSS (0.001 µM paclitaxel, 1000 µM carboplatin, 2 Gy irradiation), MAGIC (1.493 µM epirubicin, 1.5 µM cisplatin, 0.8249 µM 5-fluorouracil. (**F**) Bar chart showing the frequencies of NKp30^+^ NK cells in the circulation of non-obese (*n* = 5) and obese (*n* = 10) OAC patients by VFA. (**G**) Bar chart showing the frequency of circulating NKp30^+^ NK cells following treatment FLOT (*n* = 9), CROSS (*n* = 4) or no treatment (*n* = 4) (treatment naïve) in OAC patients at time of surgical resection. (**H**) Bar chart showing the showing the frequencies of NKp30^+^ NK cells in the circulation of OAC patients stratified by tumour regression grade TRG 1–2 (*n* = 5, good responders) and TRG 3–5 (*n* = 5, poor responders). (**I**) Bar chart showing the frequency of circulating NKG2D^+^ NK cells following treatment FLOT (*n* = 10), CROSS (*n* = 4) or no treatment (*n* = 4) (treatment naïve) in OAC patients at time of surgical resection. (**J**) Bar chart showing the frequency of circulating NKp46^+^ NK cells following treatment FLOT (*n* = 9), CROSS (*n* = 4) or no treatment (*n* = 4) (treatment naïve) in OAC patients at time of surgical resection. *p < 0.05, **p < 0.01 by One-way ANOVA with post-hoc Dunnett’s or Tukey’s test or t-test as appropriate. [Mean + SEM]

To ascertain whether significantly lower surface expression of activating NKR ligands on OE33CisR cells was due to increased shedding, a panel of soluble NKR ligands were measured in the supernatants of the OE33CisP and OE33CisR cells (B7-H6, MICA/B and ULBP3). Similar B7-H6 concentration were observed in the supernatant of OE33CisP and OE33CisR cells, with both cell lines shedding less than 0.5 pg B7-H6 per µg/ml of protein (Fig. 3A). Furthermore, there were undetectable levels of MICA and ULBP-3 soluble protein in the cell line supernatant from OE33CisP and OE33CisR cell lines [data not shown]. These data suggest that the significantly lower surface expression of B7-H6, MICA/B and ULBP-3 by OE33CisR cell lines is not due to increased shedding of these ligands.

To examine B7-H6 ligand shedding in vivo, soluble B7-H6 levels were measured in the serum of OAC patients. Interestingly, there was a significant positive correlation between visceral fat area (VFA) and serum B7-H6 protein (*r* = 0.8113, *p* = 0.0044, *n* = 10) (Fig. 3B). Interestingly, there were considerably lower frequencies of NKp30^+^ NK cells in the circulation of obese (*n* = 10) OAC patients, compared to their non-obese (*n* = 5) counterparts (Non-obese vs obese; 88.84% vs. 64.02%, *p* = 0.06) (Fig. 3F). Moreover, higher levels of serum B7-H6 were detected in OAC patients with a higher tumour regression grade TRG (TRG 3–5, *n* = 6) which is indicative of poor treatment response, compared to patients with a lower TRG (TRG 1–2, *n* = 4) (Low TRG vs. High TRG; 257.0 pg/ml vs. 967.2 pg/ml, *p* = 0.1021) (Fig. 3D). There were significantly lower frequencies of NKp30^+^ NK cells in the circulation of OAC patients who had a higher TRG (TRG 3–5, *n* = 4) compared to those with a lower TRG (TRG 1–2, *n* = 4) (Low TRG vs. High TRG; 79.63% vs. 51.55%, *p* = 0.0259) (Fig. 3H). These data suggest that OAC patients with greater VFA and poorer treatment response have higher levels of circulating B7-H6 and lower frequencies of circulating NKP30^+^ NK cells.

To determine whether treatment with neoadjuvant chemotherapy or CRT is associated with altered circulating levels of soluble B7-H6, serum B7-H6 levels were measured in OAC patients at time-points pre- and post-treatment. While there were substantially higher levels of soluble B7-H6 levels in the serum of patients post-FLOT, these were not significantly different (5-fluorouracil, oxaliplatin, docetaxel) (Pre-FLOT vs. Post-FLOT; 558 pg/ml vs. 733.8 pg/ml, *n* = 5). Furthermore, there were no significant differences observed in the soluble B7-H6 levels in the serum of patients prior to CROSS (Pre-CROSS), compared to the Post-CROSS time-point (paclitaxel, carboplatin, ± 2 Gy irradiation) (Pre-CROSS vs. Post-CROSS; 561.1 pg/ml vs. 535.68 pg/ml, *n* = 4) (Fig. 3C).

Interestingly, there were significantly lower frequencies of NKp30^+^ NK cells in the circulation of OAC patients who received FLOT (*n* = 9), compared to those who received CROSS (*n* = 4) (FLOT vs. CROSS; 48.99% vs. 86.55%, *p* = 0.0121) or were treatment naïve (*n* = 4) at the time of surgical resection (FLOT vs. Naïve; 48.99% vs. 82.4%, *p* = 0.0247) (Fig. 3H). Similarly, there were significantly less NKG2D^+^ NK cells in the circulation of OAC patients who received FLOT (*n* = 10), compared to those who received CROSS (*n* = 4) (FLOT vs. CROSS; 62.52% vs. 87.60%, *p* = 0.05) or were treatment naïve (*n* = 4) at the time of surgical resection (FLOT vs. Naïve; 62.52% vs. 90.03%, *p* = 0.0335) (Fig. 3I). Furthermore, there were significantly less NKp46^+^ NK cells in the circulation of OAC patients who received FLOT (*n* = 10), compared to those who received CROSS (*n* = 4) (FLOT vs. CROSS; 62.8% vs. 96.15%, *p* = 0.0046). There appears to be considerably less NKp46^+^ NK cells in the circulation of OAC patients who were treatment naïve (*n* = 4) at the time of surgical resection compared to those who received FLOT (FLOT vs. Naïve; 62.8% vs. 82.85%, *p* = 0.08) (Fig. 3J).

To ascertain whether treatment with clinically relevant chemotherapeutic combinations altered B7-H6 shedding into the treatment-naïve OAC tumour microenvironment, OE33 cells were treated with FLOT (5-fluorouracil, oxaliplatin, docetaxel), CROSS (paclitaxel, carboplatin, ± 2 Gy irradiation), and MAGIC (epirubicin, cisplatin, 5-fluorouracil) chemo-radiotherapy regimens. Interestingly, there was a significant increase in soluble B7-H6 in the supernatant of OE33 cells following treatment with FLOT chemotherapy regimen, compared to vehicle control (Vehicle control vs. FLOT chemotherapy; 483.6 pg/ml vs. 784 pg/ml; *p* = 0.0034) (Fig. 3E). In contrast, significant decreases in soluble B7-H6 were observed following treatment with CROSS chemotherapy (Vehicle control vs. CROSS chemotherapy; 483.6 pg/ml vs. 177.8 pg/ml, *p* = 0.0031), and CROSS chemoradiotherapy (Vehicle control vs. CROSS chemoradiotherapy; 483.6 pg/ml vs. 221 pg/ml; *p* = 0.0077) (Fig. 3E).

### Significantly lower frequencies of NK cells expressing activating NKRs following co-culture with OE33CisP and OE33CisR cells

**Fig. 4:**
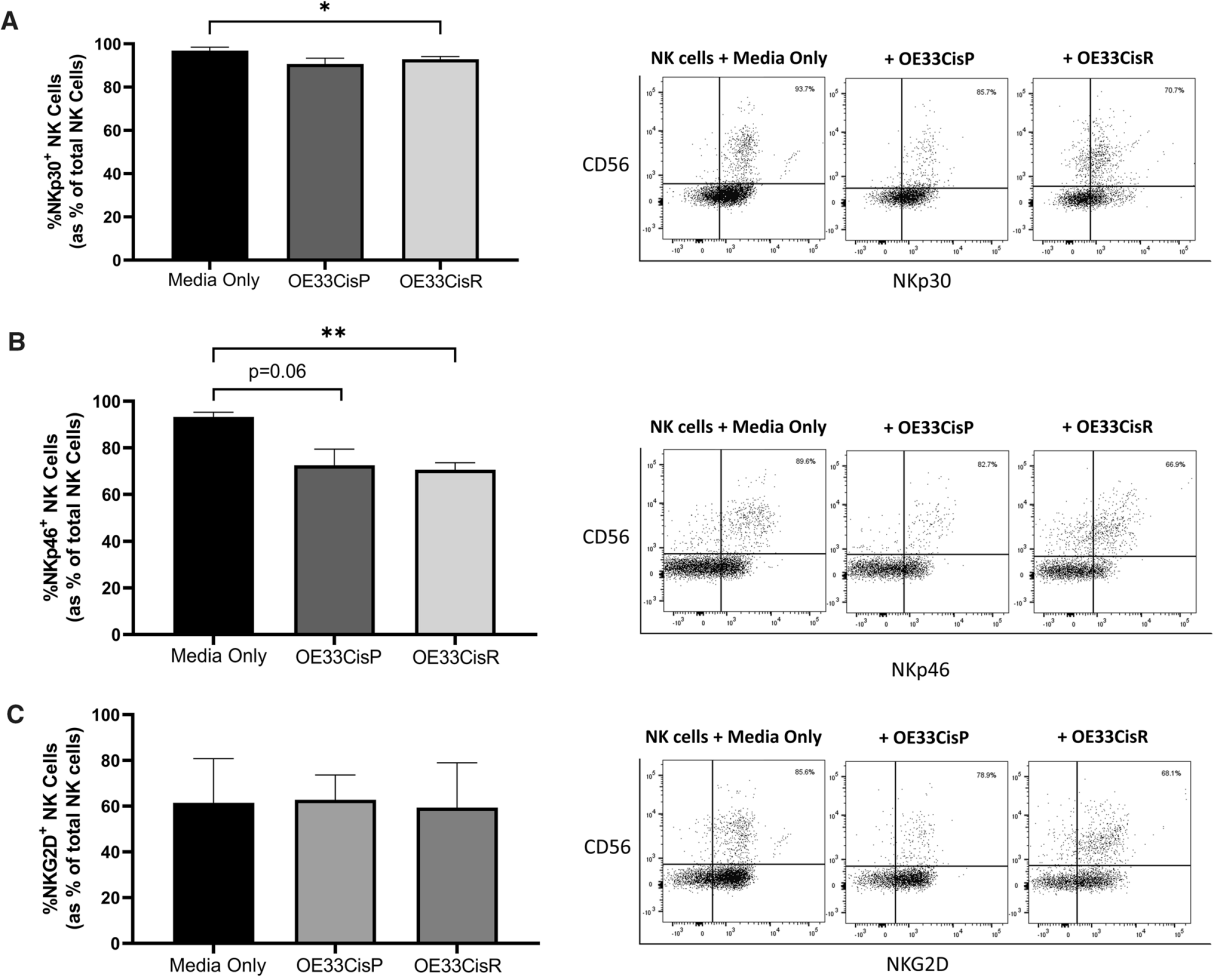
Significantly lower frequencies of NK cells expressing activating receptors following culture with cisplatin-resistant oesophageal cancer cells. (**Left**) Bar chart showing the frequencies of (**A**) NKp30^+^, (**B**) NKp46^+^ and (**C**) NKG2D^+^ NK cells following media only or co-culture with OE33CisP or OE33CisR cells (*n* = 3–5). (**Right**) Representative dot plots showing (**A**) NKp30^+^, (**B**) NKp46^+^ and (**C**) NKG2D^+^ NK cells previously gated on total lymphocytes. Percentage frequencies shown are those of (**A**) NKp30^+^, (**B**) NKp46^+^ and (**C**) NKG2D^+^ NK cells as percentage of total NK cells. *p < 0.05, **p < 0.01 by one-way ANOVA with post-hoc Dunnett’s test. [Mean + SEM]

To elucidate the effects of cisplatin-resistant OAC tumours on the phenotype of NK cells, proportions of cells expressing activating NKRs were assessed following co-culture with OE33CisP or OE33CisR cells. Our data revealed lower frequencies of NKp30^+^ NK cells following co-culture with OE33CisR cells compared to NK cells cultured alone (*n* = 3; Media only vs. OE33CisR; 96.87% vs. 92.87%; *p* = 0.0181) (Fig. 4A). Similarly, there were significantly lower frequencies of NKp46^+^ NK cells following co-culture with OE33CisR cells compared to NK cells cultured alone (*n* = 5; Media only vs. OE33CisR; 93.26% vs. 70.64%; *p* = 0.0020) (Fig. 4B). It appears that co-culture with the cisplatin-sensitive OE33CisP cells also decreased the frequencies of NKp46^+^ NK cells, compared to those left untreated (*n* = 5; Media only vs. OE33CisP; 93.26% vs. 72.56%; *p* = 0.06) (Fig. 4B). There were no significant differences in the frequencies of NKG2D^+^ NK cells following co-culture with OE33CisP or OE33CisR cells (Fig. 4C).

### Significantly higher frequencies of NK cells expressing TIGIT following co-culture with OE33CisR cells

**Fig. 5:**
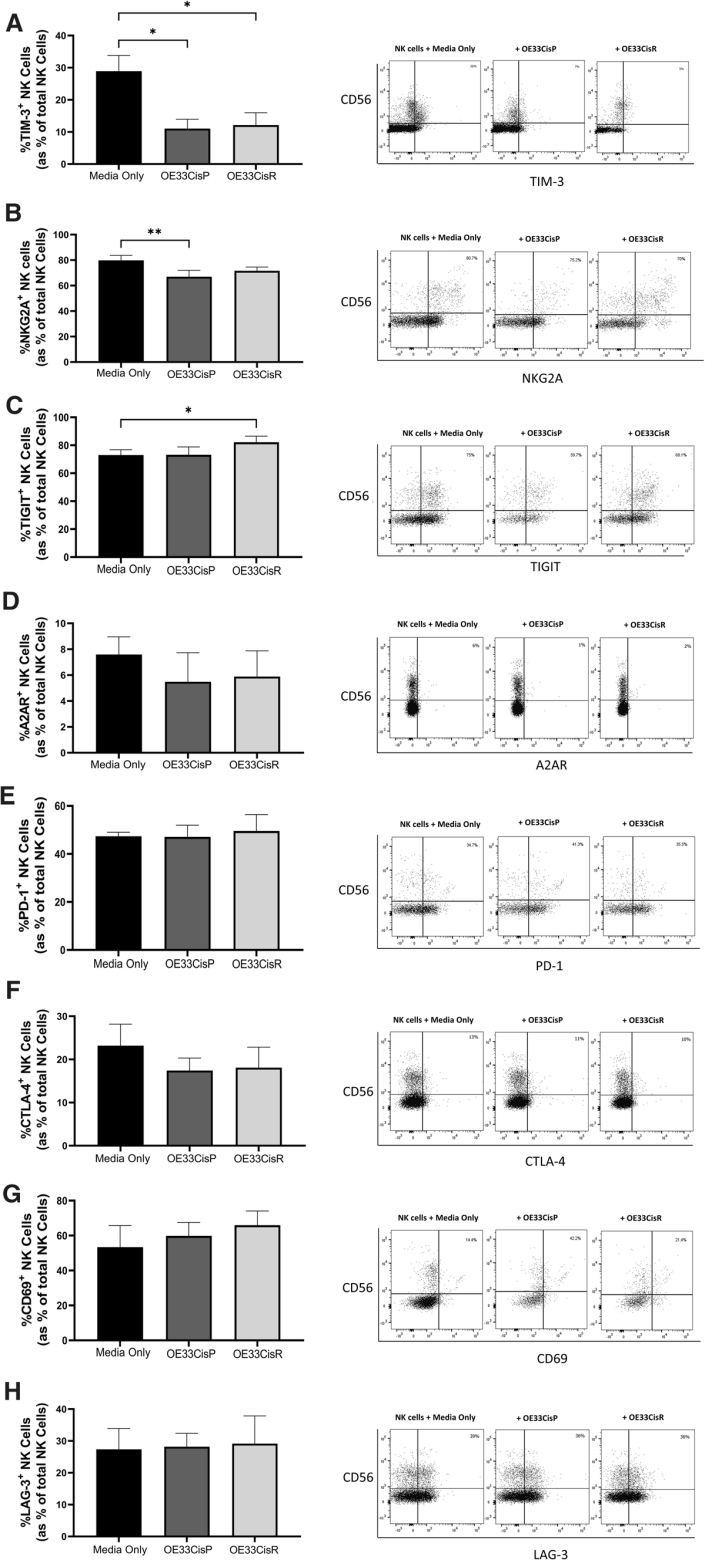
Significantly lower frequencies of TIM-3^+^ NK cells following co-culture with OE33Cisp or OE33CisR cells while frequencies of TIGIT^+^ NK cells are higher following culture with OE33CisR. (**Left**) Bar chart showing the frequencies of (**A**) TIM-3^+^, (**B**) NKG2A^+^, (**C**) TIGIT^+^, (**D**) A2AR^+^, (**E**) PD-1^+^, (**F**) CTLA-4^+^, (**G**) CD69^+^ and (**H**) LAG-3^+^ NK cells following culture alone or co-culture with OE33CisP or OE33CisR cells (*n* = 3). (**Right**) Representative dot plots showing (**A**) TIM-3^+^, (**B**) NKG2A^+^, (**C**) TIGIT^+^, (**D**) A2AR^+^, (**E**) PD-1^+^, (**F**) CTLA-4^+^, (**G**) CD69^+^ and (**H**) LAG-3^+^ NK cells previously gated on total lymphocytes. Percentage frequencies shown are those of (**A**) TIM-3^+^, (**B**) NKG2A^+^, (**C**) TIGIT^+^, (**D**) A2AR^+^, (**E**) PD-1^+^, (**F**) CTLA-4^+^, (**G**) CD69^+^ and (**H**) LAG-3^+^ NK cells as percentage of total NK cells. *p < 0.05, **p < 0.01 by one-way ANOVA with post-hoc Dunnett’s test. [Mean + SEM]

The frequencies of NK cells expressing the immune checkpoint TIM-3 were significantly lower following co-culture with OE33CisP (*n* = 3, media only vs. OE33CisP; 28.9% vs. 11.03%, *p* = 0.0334) and CisR cells (*n* = 3, media only vs. OE33CisR; 28.9% vs. 12%, *p* = 0.0428) compared to those cultured alone (Fig. 5A). Following co-culture of NK cells with OE33CisP cells, the frequencies of NK cells expressing the inhibitory receptor NKG2A was significantly decreased compared to NK cells cultured alone (*n* = 5; media only vs. OE33CisP; 79.78% vs. 66.98%, *p* = 0.0082) (Fig. 5B). Interestingly, there were significantly higher frequencies of TIGIT^+^ NK cells following co-culture with the cisplatin-resistant OE33CisR cells, compared to NK cells cultured alone (*n* = 5, media only vs. OE33CisR; 73.06% vs. 82.20%; *p* = 0.0421) (Fig. 5C). No changes were observed in the frequencies of A2AR^+^, PD-1^+.^, CTLA-4^+^, LAG-3^+^ or CD69^+^ NK cells following co-culture with OE33CisP or OE33CisR cells (Fig. 5D–H).

### Significantly higher frequencies of NK cells expressing the death receptor ligands TRAIL and FasL following culture with cisplatin-resistant OAC cells

**Fig. 6:**
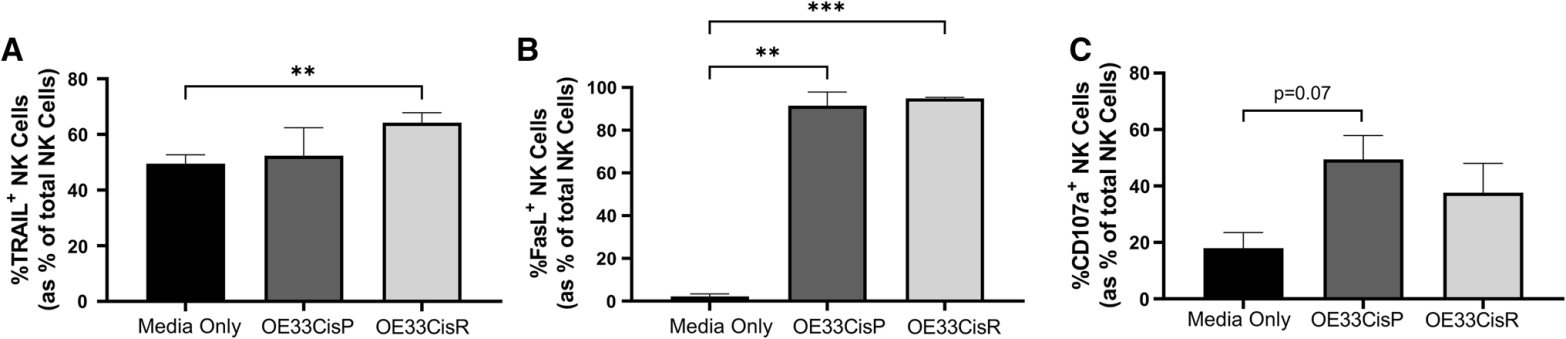
Significantly higher frequencies of NK cells expressing death receptor ligands following co-culture with cisplatin-resistant OE33 cells. Bar chart showing the frequencies of (**A**) FasL^+^, (**B**) TRAIL^+^ and (**C**) CD107a^+^ NK cells following culture alone or co-culture with OE33CisP or OE33CisR cells (*n* = 3). **p < 0.01, ***p < 0.001 by one-way ANOVA with post-hoc Dunnett’s test. [Mean + SEM]

There were significantly higher frequencies of TRAIL^+^ NK cells following co-culture with OE33CisR cells, compared to NK cells cultured alone (*n* = 3; media only vs. OE33CisR; 49.50% vs. 64.23%; *p* = 0.0019) (Fig. 6A). Furthermore, there were significantly higher frequencies of NK cells expressing the death receptor ligand FasL following culture with both OE33CisP (*n* = 3; media only vs. OE33CisP; 2.203% vs. 91.43%; *p* = 0.0065) and OE33CisR cells (media only vs. OE33CisR; 2.203% vs. 94.83%; *p* = 0.0005) (Fig. 6B). While not significant, the ability of NK cells to degranulate, as indicated by CD107a expression, may be increased following co-culture with OE33CisP cells (*n* = 3–5; media alone vs. OE33CisP; 14.79% vs. 49.40%; *p* = 0.07) (Fig. 6C).

### NK cell cytokine production is significantly altered following co-culture with OAC cells

**Fig. 7:**
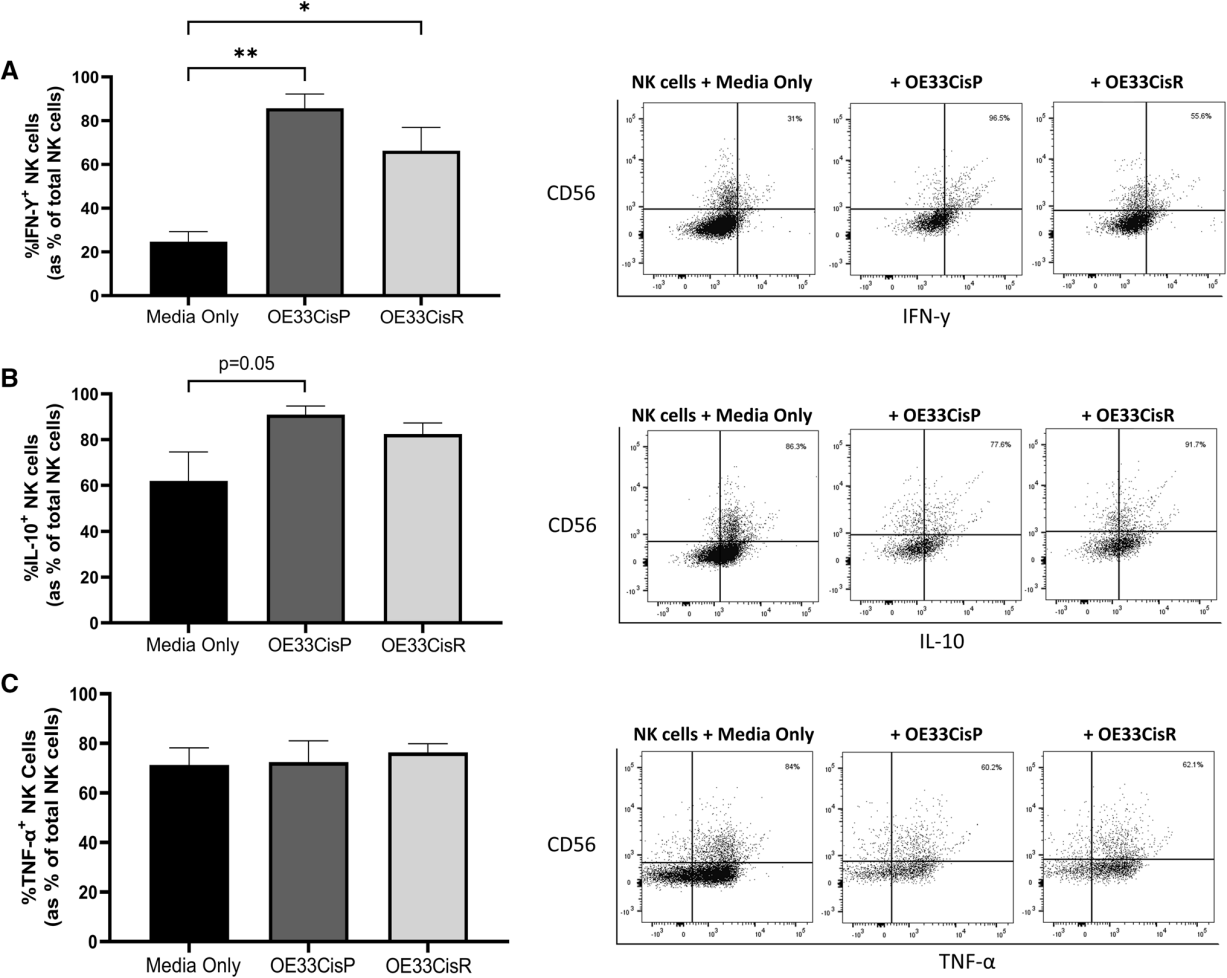
Significantly higher frequencies of NK cells producing IFN-*γ* following co-culture with OAC cells. (**Left**) Bar chart showing the frequencies of (**A**) IFN-*γ*^+^, (**B**) IL-10^+^ and (**C**) TNF-*α*^+^ NK cells following culture alone or co-culture with OE33CisP or OE33CisR cells (*n* = 3). (**Right**) Representative dot plots for (**A**) IFN-*γ*^+^, (**B**) IL-10^+^ and (**C**) TNF-*α*^+^ NK cells previously gated on total lymphocytes. Percentage frequencies shown are those of (**A**) IFN-*γ*^+^, (**B**) IL-10^+^ and (**C**) TNF-*α*^+^ NK cells as percentage of total NK cells. *p < 0.05, **p < 0.01 by one-way ANOVA with post-hoc Dunnett’s test. [Mean + SEM]

To elucidate the functional effects of co-culture with OE33CisP and OE33CisR cells on NK cells, NK cell production of the pro-inflammatory cytokines IFN-*γ* and TNF-*α*, and the anti-inflammatory cytokine IL-10 was examined. Interestingly, while there are significantly more IFN-*γ*^+^ NK cells following co-culture with OE33CisP (*n* = 3, media alone vs. OE33CisP; 24.67% vs. 85.73%; *p* = 0.0100) and OE33CisR cells (*n* = 6, media alone vs. OE33CisR; 24.67% vs. 66.25%; *p* = 0.0336) compared to those cultured alone, the increases in IFN-*γ*^+^ NK cells are considerably lower when cultured with the resistant cells, compared to the sensitive cells (Fig. 7A). The frequencies of IL-10^+^ NK cells were also increased following co-culture with OE33CisP cells, compared to those cultured alone (*n* = 3, media alone vs. OE33CisP; 61.93% vs. 90.97%; *p* = 0.05) (Fig. 7B). There were no changes in the frequencies of TNF-*α* producing NK cells following co-culture with OE33CisP or OE33CisR cells (Fig. 7C).

## Discussion

Previous work by our group has uncovered altered NK cell frequencies and function in OAC patients and we propose that NK cell therapy might be utilised to restore NK cell-mediated anti-tumour immunity for this cohort, who face dismal survival rates and a paucity of treatment options [16, 17]. However, with advances in NK cell-based immunotherapies, it is prudent to be aware that many OAC patients will have previous exposure to standard-of-care treatments and an overwhelming majority of OAC patients may have treatment-resistant tumours. As such, it is imperative that we characterise these tumours to assess their susceptibility and suitability for NK cell-based therapies.

To gauge susceptibility to NK cell-mediated responses and indeed to NK cell therapy, we characterised the NKR ligand expression profile of our in-house cisplatin-resistant OAC cell line model OE33CisR and its cisplatin-sensitive counterpart, OE33CisP. Here, we identified significantly lower surface expression of NKR activating ligands B7-H6, MICA/B and ULBP-3 and activating/inhibitory NKR ligands PVRL-1 and PVRL-4 on OE33CisR cells compared to cisplatin-sensitive OE33CisP cells. Such diminished NKR ligand expression suggests that cisplatin-resistant OAC tumours may be less susceptible to NK cell-mediated killing than their cisplatin-sensitive counterparts.

Downregulation in gene expression of the NKG2D ligands, ULBP-1, ULBP-3 and MICA/B has been shown in cisplatin-resistant lung cancer cell lines compared to their parental counterparts, with mitogen-activated protein kinase kinase/extracellular signal-regulated kinase (MAPK/Erk) signalling implicated in this reduced ligand expression [18]. We hypothesised that the differences observed in NKR ligand surface expression between OE33CisP and OE33CisR may be due to alterations at the gene expression level. Our data however showed no significant differences in the levels of B7-H6, HLA-E, PVRL-1, PVRL-4 and ULBP3 mRNA expression between OE33CisP and OE33CisR cells.

While there has been success in utilising cellular based immunotherapies in the treatment of haematological malignancies, solid tumours pose additional challenges [19]. The solid tumour microenvironment (TME) is one such challenge and identifying the factors within the TME that may contribute to the reduced efficacy of cellular therapies is imperative to ensuring their success. Cleavage of NK activating ligands B7-H6 and MICA/B expressed on tumour cells and the subsequent release of these soluble ligands is a strategy through which tumour cells evade NK cell-mediated immune surveillance and thus may dampen the effects of NK cell therapies [20–22]. Since NKR ligands were not altered at a transcriptional level, we proposed that ligands were expressed but subsequently shed into the TME of treatment-resistant tumour cells. Our in vitro data did not support this hypothesis and the lower surface expression of B7-H6, MICA/B and ULBP-3 by OE33CisR cells was not paralleled by significantly higher soluble NKR ligand levels in the OE33CisR cell supernatant.

B7-H6 is an activating ligand for the receptor NKp30 and has gained much traction as a potential prognostic marker in multiple malignancies [23]. It is expressed preferentially on tumour cells, thus centring it as an important target for NK cell based therapies [23]. Interaction of NKp30 with its ligand B7-H6 can modulate the downregulation of NKp30 on the surface of NK cells [24]. Soluble B7-H6 has been shown to contribute to defective expression of NKp30 on circulating NK cells from hepatocellular carcinoma, ovarian carcinoma and neuroblastoma patients, further linking elevated B7-H6 with NK cell dysfunction [24–26]. Indeed, soluble B7-H6 has been proposed as a biomarker for several cancers. In the case of gastrointestinal stromal tumours, detectable levels of serum B7-H6 were predictive of a poor treatment response and worse outcome for patients [27]. In neuroblastoma, B7-H6 levels inversely correlate with NKp30 surface expression suggesting that B7-H6 can act in the periphery by engaging the NKp30 receptor and thus reducing NKp30-dependent NK cell functions [28]. B7-H6 expression correlated with unfavourable prognosis for T-lymphoblastic lymphoma patients [29]. In patients with acute myeloid leukaemia, an NKp30^high^ phenotype was predictive of a better outcome for patients [30].

Interestingly, our clinical analysis of patient serum samples from treatment responders and non-responders revealed the highest levels of soluble B7-H6 in the serum of OAC patients with a higher tumour regression grade TRG (TRG 3–5) following CRT, which is indicative of poor treatment response. These trends would suggest that poor treatment responders have higher circulating levels of soluble B7-H6, which might be indicative of higher intratumoural levels and a possible immune evasion strategy within treatment-resistant OAC tumours. The higher levels of circulating soluble B7-H6 in poor treatment responders were paralleled by significantly lower frequencies of circulating NK cells expressing its cognate receptor, NKp30. NKp30^low^ NK cells have been shown to exhibit impaired cytolytic function and IFN-*γ* production thus suggesting that OAC patients who are poor treatment responders have diminished NK cell activity, possibly mediated by enhanced levels of soluble B7-H6 [25].

There is compelling evidence to suggest chemotherapeutic agents modulate cellular stress pathways, and could impact NK cell expression of activating and inhibitory ligands [31]. Determining the chemotherapeutic effects on NKR ligand shedding by tumour cells can inform the timing of NK cell therapies with first-line treatments in OAC. To ascertain whether chemotherapeutic combination regimens used to treat OAC could alter NKR ligand shedding in the TME, treatment-naïve OAC tumour cells were treated with FLOT, CROSS and MAGIC [32]. Our results demonstrate that CROSS can reduce B7-H6 shedding in OE33 cells. This places CROSS as a preferable regimen to potentially combine with NK cell therapies to minimise immune evasion strategies mediated through B7-H6 shedding. In contrast, the FLOT regimen induced B7-H6 shedding by OE33 cells, hence this regimen may hinder NK cell responses and indeed NK cell therapy. Indeed, here we report significantly lower frequencies of NKp30^+^ NK cells in the circulation of OAC patients who received the FLOT treatment regimen compared to those who received the CROSS regimen or were treatment naïve at surgical resection. This is paralleled by significantly lower frequencies of NKG2D^+^ and NKp46^+^ NK cells in OAC patient blood following treatment with FLOT compared to CROSS or no treatment. Exposure to soluble B7-H6 has been shown to cause a decrease in expression of these activating receptors on NK cells, indicating B7-H6 has wide reaching effects beyond its own cognate receptor [33]. Interestingly, the chemotherapeutic 5-FU which is a component of the FLOT regimen has been shown to increase B7-H6 expression on tumour cells [34]. Furthermore, 5-FU is also a known activator of ADAM17, the sheddase which causes the shedding of B7-H6 from the tumour cell surface [35]. This suggests that FLOT chemotherapy regimen may both increase the expression of B7-H6 on the tumour cell surface and aid in mediating the shedding of B7-H6 into the tumour microenvironment which may lead to defective NK cell function.

Interestingly, serum levels of B7-H6 were not significantly altered following first line treatments in OAC patients and circulating B7-H6 levels were not significantly higher following FLOT. Since such post-CRT serum samples were collected on the day of surgery and 6 weeks after last dose of chemotherapy, these observations suggest that either the stimulatory or inhibitory effects of chemotherapy on B7-H6 shedding are not maintained after treatment has ceased, or that the serum may not be a useful indicator of chemotherapy-induced NKR ligand shedding within the TME of OAC patients. Overall our data suggest that analysis of NKp30 expression on circulating NK cells may be superior to serum B7-H6 as a blood biomarker for treatment response and may allow for the identification of OAC patients who are treatment-resistant. Furthermore, NKP30 may be used to inform the use of chemoradiotherapy regimens which do not promote an inhibitory NK cell phenotype and thus would be more appropriate to pair with an NK cell-based immunotherapy.

Interestingly, here we report a significant correlation between increasing visceral adiposity and B7-H6 serum levels in OAC patients. Furthermore, frequencies of NK cells expressing the activating receptor NKp30 were significantly lower in obese OAC patients, compared to their non-obese counterparts, as stratified by VFA. OAC has a strong association with obesity, and we have reported extensive alterations in both the phenotype and function of NK cells in these obesity-associated cancer patients, with the most obese patients having the lowest number of intratumoural NK cells [16, 17, 36]. This present study suggests that NKR ligand shedding is exacerbated in viscerally obese OAC patients and may contribute to diminished NKp30 expression on circulating NK cells and compromised anti-tumour responses in these patients. These data provide further evidence that visceral obesity is an important pathological entity with wide-reaching effects [37].

Despite no obvious differences in the shedding of several key NKR ligands in our cell line models of cisplatin-resistant OAC tumours, our data revealed that co-culture with OE33CisP and OE33CisR cells could significantly modulate surface expression of NKRs on primary human NK cells. The frequencies of NKp46^+^ and NKp30^+^ NK cells were significantly lower following co-culture with OE33CisR cells. Soluble factors such as TGF-β, activin-A and adenosine are known to inhibit NK cell activity and mediate tumour evasion and thus may be contributing to these alterations in NK cell phenotype following co-culture with the cisplatin-resistant OAC cells [38]. These results demonstrate that treatment-resistant OAC tumour cells can decrease the frequencies of NK cells expressing activating receptors, and thus may hinder recognition of tumour cells and NK cell-mediated killing. A shift in the phenotype of NK cells via alterations in NKR expression has previously been reported to dampen immune surveillance and cytotoxic abilities of NK cells, hence facilitating tumour growth in ovarian cancer [39]. While the mediators of such reductions were not identified in this study, our group have previously reported that OE33CisR cells have altered inflammatory chemokines and cytokines profiles [13]. In particular, the production of key NK cell cytokines IL-2, IL-10 and TNF-*α* is significantly lower from OE33CisR cells compared to their OE33CisP counterparts [13].

Our data also revealed that frequencies of immune checkpoint TIGIT^+^ NK cells were increased following culture with OE33CisR cells. Interestingly, the frequencies of NK cells expressing the inhibitory receptor NKG2A were significantly decreased following co-culture with OE33CisP cells, whilst their frequencies were maintained following co-culture with the cisplatin-resistant OE33CisR cells. High expression of surface TIGIT contributes to NK cell exhaustion in solid tumours and therefore blockade can enhance NK cell-mediated responses [40, 41]. Combination of TIGIT blockade with the anti-human epidermal growth factor receptor 2 antibody trastuzumab has been shown to enhance NK cell-mediated antibody-dependent cellular cytotoxicity [42]. These data provide new evidence that targeting the immune checkpoints TIGIT may be particularly effective in the setting of treatment-resistant OAC tumours. The frequencies of TIM-3^+^ NK cells were significantly decreased following culture with both OE33CisP cells and OE33CisR cells. TIM-3 is a known NK cell maturation marker which possesses both activating and inhibitory functions and has been shown to both impair NK cell mediated cytotoxicity and enhance IFN-*γ* production [43, 44]. In line with results reported here, exposure to glioblastoma and prostate cancer cells resulted in a downregulation of TIM-3 [45]. Interestingly, while previous studies have reported a correlation between TIM-3 expression and decreased cytotoxicity, we report significantly higher frequencies of death receptor ligand and IFN-*γ* expressing NK cells following co-culture with both cell lines, suggesting TIM-3 mediated effects on these markers are not immediate [45]. Furthermore, it suggests in this setting that TIM-3 may have an inhibitory role for NK cells in OAC and that cancer cell mediated decreases in the expression of this marker may facilitate enhanced NK cell function in both the cisplatin-sensitive and resistant OAC tumour. The multi-faceted role of TIM-3 in relation to NK cell phenotype and function in OAC warrants further exploration.

Despite decreases in activating NKR expression following co-culture with OE33CisR cells, our data revealed that frequencies of death receptor ligands TRAIL^+^ and FasL^+^ NK cells were significantly increased. These data suggest that the OAC TME promotes the upregulation of key death receptor ligands on the surface of NK cells and provides a glimpse into the potential mechanisms NK cells employ to elicit their effects against such tumours. Furthermore, our findings uncovered significantly higher frequencies of pro-inflammatory IFN-*γ*^+^ NK cells following culture with both OE33CisP and OE33CisR cells, further suggesting that the cisplatin-resistant OAC tumour does not significantly impede NK cell function. Indeed, NK cell-derived IFN-*γ* plays a pivotal role in tumour cell cytolysis via promotion of conjugate formation [46] and our group has previously reported that the OAC tumour is enriched with this pro-inflammatory cytokine, which has been shown to mediate death receptor ligand expression [47, 48]. Additionally, it appears that neither cisplatin-sensitive nor cisplatin-resistant OAC tumour cells hamper NK cell degranulation, as indicated by CD107a expression and may even increase it. Overall, these data suggest that the introduction of NK cells to the OAC tumour microenvironment does not irrevocably alter NK cell function and that such tumours may be amenable to NK cell therapies.

## Conclusions

In this study, we report for the first time, the profiling of NKR ligand expression in cisplatin-sensitive and cisplatin-resistant OAC cells, identifying unique ligand expression profiles in this cancer type. While co-culture with treatment-resistant OAC tumour cells alters the frequencies of NK cells expressing activating and inhibitory NKRs, immune checkpoints and death receptors, they do not negatively modulate IFN-*γ* production or degranulation of these potent anti-tumour cells. Such altered NKR profiles are mirrored in OAC patients in which poor treatment responders have significantly lower numbers of the key NKP30^+^ NK cell subset. Interestingly, we have identified that the FLOT chemotherapy regimen induces NKP30 ligand B7-H6 shedding by OAC tumour cells and thus their subsequent susceptibility to NK cells and NK cell-based therapies. In line with this, the frequencies of NK cells expressing NKp30 are significantly diminished following FLOT chemotherapy in OAC patients. Finally our data identify that B7-H6 serum levels correlate with visceral adiposity suggesting that obesity exacerbates such ligand shedding and the resulting NK cell modulation. As such, it is integral that the impact of both first-line treatments and the obese environment are considered for the successful design of NK cell-based therapies.

## Declarations

### Availability of data and material

Data sharing not applicable to this article as no datasets were generated or analysed during the current study.
